# Naturally selected CD7 CAR-T therapy without genetic editing demonstrates significant antitumour efficacy against relapsed and refractory acute myeloid leukaemia (R/R-AML)

**DOI:** 10.1186/s12967-022-03797-7

**Published:** 2022-12-14

**Authors:** Yu Lu, Ying Liu, Shupeng Wen, Na Kuang, Xuejun Zhang, Jianqiang Li, Fuxu Wang

**Affiliations:** 1 Department of Hematology, Key Laboratory of Hematology of Hebei Province, Second Hospital of Hebei Medical University, Shijiazhuang, China; 2 Hebei Senlang Biotechnology Co, Shijiazhuang, China

**Keywords:** Acute myeloid leukemia_1_, CD7_2_, CAR-T cells_3_, Immunotherapy_4_, Minimal Residual disease (MRD)_5_

## Abstract

**Background:**

The survival rate for patients with relapsed and refractory acute myeloid leukaemia (R/R-AML) remains poor, and treatment is challenging. Chimeric antigen receptor T cells (CAR-T cells) have been widely used for haematologic malignancies. Current CAR-T therapies for acute myeloid leukaemia mostly target myeloid-lineage antigens, such as CD123 and CD33, which may be associated with potential haematopoietic toxicity. As a lineage-specific receptor, CD7 is expressed in acute myeloid leukaemia cells and T cells but is not expressed in myeloid cells. Therefore, the use of CD7 CAR-T cells for R/R-AML needs to be further explored.

**Methods:**

In this report, immunohistochemistry and flow cytometry were used to analyse CD7 expression in clinical samples from R/R-AML patients and healthy donors (HDs). We designed naturally selected CD7 CAR-T cells to analyse various functions and in vitro antileukaemic efficacy based on flow cytometry, and xenograft models were used to validate in vivo tumour dynamics.

**Results:**

We calculated the percentage of cells with CD7 expression in R/R-AML patients with minimal residual disease (MRD) (5/16, 31.25%) from our institution and assessed CD7 expression in myeloid and lymphoid lineage cells of R/R-AML patients, concluding that CD7 is expressed in T cells but not in myeloid cells. Subsequently, we designed and constructed naturally selected CD7 CAR-T cells (CD7 CAR). We did not perform CD7 antigen knockdown on CD7 CAR-T cells because CD7 molecule expression is naturally eliminated at Day 12 post transduction. We then evaluated the ability to target and kill CD7^+^ acute myeloid leukaemia cells in vitro and in vivo. Naturally selected CD7 CAR-T cells efficiently killed CD7^+^ acute myeloid leukaemia cells and CD7^+^ primary blasts of R/R-AML patients in vitro and significantly inhibited leukaemia cell growth in a xenograft mouse model.

**Conclusion:**

Naturally selected CD7 CAR-T cells represent an effective treatment strategy for relapsed and refractory acute myeloid leukaemia patients in preclinical studies.

## Background

Acute myeloid leukaemia (AML) is a malignancy characterized by abnormal clonal expansion of myeloid blasts in the bone marrow that impairs normal haematopoiesis, causing infection, bleeding, and anaemia [1]. Despite standard treatment with chemotherapeutic agents, including anthracycline and cytarabine, to achieve complete remission (CR) for patients with acute myeloid leukaemia, many patients still relapse within a short period [2, 3]. Only 35–45% of patients under the age of 60 years experience long-term remission with conventional treatment, and the proportion drops to 10–15% for those older than 60 years [2, 4]. Relapse and leukaemia-related complications are the most common causes of death, and only 10% of patients with a first relapse survive long term [4, 5].

In recent years, cellular immunotherapy of chimeric antigen receptor T cells (CAR-T) for relapsed or refractory acute myeloid leukaemia (R/R-AML) targeting myeloid-lineage antigens, such as CD123, CLL-1, and CD33, has shown promising prospects in many preclinical studies [6–11]. CD123 and CD33 expression in CD34^+^ haematopoietic stem cells and other normal myeloid cells poses a potential haematopoietic risk. In addition, evidence for long-term remission is lacking, so safety and efficacy need further evaluation [12–14].

Selecting lineage-specific antigens specifically expressed on AML cells but not expressed on normal myeloid cells may represent a promising therapeutic strategy. CD7 is a target that has been used in the therapeutic strategy of peripheral T-cell leukaemia with favourable results in preclinical and clinical studies [15–17]. Approximately 20–35% of AML exhibits high CD7 expression, which is frequently associated with a worse patient prognosis [18–22]. Under physiological conditions, CD7 is expressed on NK cells, T cells and lymphoid progenitor cells [23–27]. It is involved in the positive regulation of T-cell activity, but its deletion does not interfere with T-cell development and function because the T-cell function of CD7 knockout mice is not impaired [28, 29]. Recent clinical trials have also demonstrated that the proliferation of CD7^−^ T-cells after CD7^+^ T-cell depletion in patients may compensate for the immune deficiency due to T-cell absence [30]. These pieces of evidence demonstrate the feasibility of CD7 as a target for CAR-T cells in the treatment of relapsed and refractory acute myeloid leukaemia (R/R AML).

Current studies have shown that CD7 gene-edited (CD7KO) and CD7-directed CAR-T cells have made great progress in the treatment of haematological tumours [31–33]. We propose a new research strategy regarding the use of CD7 CAR-T cells for haematologic tumours. In another study, we demonstrated in vitro, in vivo, and clinical studies that naturally selected CD7 CAR-T cells can produce promising therapeutic effects in the treatment of T-cell acute lymphoblastic leukaemia/lymphoblastic lymphoma (T-ALL/LBL) [34].

In this experiment, we demonstrated no difference in CD7 expression between the normal cell population of R/R-AML patients and healthy donors (HDs), whereas 31.25% (5/16) of R/R-AML patients exhibited varying degrees of CD7 expression. Subsequently, naturally selected CD7 CAR-T cells were constructed. Although the proliferation ability of naturally selected CD7 CAR-T cells was reduced, their transduction rate remained high, and weak CD7 was noted. However, the central memory phenotype was significantly increased compared with that noted in the NTR group. Moreover, naturally selected CD7 CAR-T cells showed rapid antileukaemia efficacy in vitro and in a CD7^+^ AML xenograft mouse model. These results indicate the feasibility of a naturally selected CD7 CAR-T cells in the treatment of CD7^+^ R/R-AML at the preclinical stage.

## Materials and methods

### Immunohistochemistry

Bone marrow biopsy samples of 3 patients with R/R-AML were obtained from the Department of Haematology, Second Hospital of Hebei Medical University. Bone marrow biopsy samples were fixed in 4% formalin (Sigma‒Aldrich, USA) at room temperature, subsequently decalcified, dehydrated, and embedded in paraffin to generate 3-µm thick tissue sections. The following immunohistochemical antibodies and reagents were obtained from Leica Microsystems (Leica, Germany). Tissue sections (3 µm thick) were subject to CD34, CD117, CD7, and MPO labelling using a Leica Bond MAX Immunostainer (Leica, Germany). The following program was employed: peroxide block for 5 min, marker labelling incubation for 15 min, post primary incubation for 8 min, polymer incubation for 8 min, DAB Refine reagent incubation for 10 min and finally haematoxylin incubation for 5 min. All the above steps were performed at room temperature. A microscope (× 400, × 100) (Olympus, Japan) was used to observe the samples.

### CD7 CAR-T-cell design and generation

The design and production of naturally selected CD7 CAR-T cells was performed as previously described by our group [34]. The DNA sequence of the anti-CD7 single-chain variable fragment derived from the CD7-specific mouse monoclonal antibody TH-69 was synthesized and cloned into a CAR composed of a 12-AA short hinge-only domain, CD28 transmembrane, 4-1BB, CD3ζ, T2A autocleavage sequences, and endodomain-deleted EGFR (tEGFR) [35–37]. CD7 CAR using lentiviral vectors was produced by transfecting and concentrating 293FT cells with 20,000 g ultracentrifugation after 2 h. The lentivirus loaded with CD7 CAR was stored at – 80 [38].

Healthy human peripheral blood mononuclear cells (PBMCs) were isolated from the peripheral blood by Ficoll density centrifugation. CD3^+^ T cells were obtained from healthy human PBMCs using CD3 microbeads (Miltenyi Biotec, Germany) and then activated for 48 h using CTS CD3/CD28 Dynabeads (Gibco, USA) in TexMACS (Miltenyi Biotec, Germany) supplemented with 200 IU of IL-2 (Sigma‒Aldrich, USA). Two days later, the CD3^+^ T cells were transduced with the CD7 CAR lentivirus. Three days after transduction, the proportion of CAR-positive (CAR^+^) T-cells was measured by flow cytometry. CD7 CAR-T-cells were continuously cultured in TexMACS (Miltenyi Biotec, Germany) with 200 IU/ml IL-2 (Sigma‒Aldrich, USA) until Day 10 post-transduction, and various in vitro functional assays or in vivo injections of CD7 CAR-T cells were performed. Initial culture numbers of CD7 CAR-T cells were increase for injection.

### Flow cytometry

The following antibodies were sourced from BioLegend, USA. Harvested cells were washed twice with DPBS (BI, Israel) with 2% FBS (Oricell, China) and subsequently incubated with labelled fluorescently conjugated antibodies (anti-human CD7-APC, CD4-PE-cy7, CD8-PB, CD3-APC-cy7, PD-1-PB, TIM-3-PE, LAG-3-APC, CD45RO-Percp, CCR7-PE, and ERB-FITC) for 20 min at 4 °C in the dark according to the assay schedule. The percentage of CD7 CAR-T cells was determined by biotinylated Erbitux (ERB) [34, 37]. Finally, all flow cytometry data were obtained in a MACSquant (Miltenyi Biotec, Germany) and analysed with FlowJo software (V10.8.1).

### CD7 expression assay

The following experimental flow cytometry antibodies were purchased from BD Bioscience, USA. All bone marrow samples were obtained from the Department of Hematology, Hebei Medical University. Bone marrow blood from patients with R/R-AML was analysed for CD45-KO, CD117-PE, CD34-APC, and CD7-A700 by flow cytometry. When levels greater than those noted the isotype control group were observed, the expression was considered positive. Then, specimens from HDs and R/R-AML patients were labelled with CD45-V500, CD14-APC, CD16-FITC, CD3-FITC, CD19-APC, and CD7-PE, and the proportion cells with CD7 expression in normal cell populations was detected by flow cytometry (Beckman Navios, USA). Finally, Kaluza software (Beckman Coulter, USA) was used to analyse flow cytometry data.

### Cytotoxicity assay

The cell lines KG-1a, MOLM-13, CCRF-CEM, and K562 were purchased from ATCC. These cells were expanded according to ATCC recommendations. A lentiviral vector was employed to overexpress CD7 in K562 cells, and K562-CD7 cells were obtained. First, target cells were added to CFSE working solution (Sigma‒Aldrich, USA) and incubated for 20 min at 37 °C in the dark. Subsequently, a total of 1 × 10^5^ KG-1a, MOLM-13, CCRF-CEM, K562, and CD7-overexpressing K562 (K562-CD7) cells were cocultured with CD7 CAR-T cells or nontransduced T cells in RPMI 1640 media (Gibco, USA) for 4 h according to the various effector and target ratios (E: T). At the end of the four-hour coculture, all cells were stained for 7-AAD-PerCP and annexin-V-APC (BioLegend, USA) and then analysed by flow cytometry (Miltenyi Biotec, Germany).

PBMC samples from 3 R/R-AML patients from the Department of Hematology, Second Hospital of Hebei Medical University were used for the primary AML cell cytotoxicity assay. Naturally selected CD7 CAR-T or nontransduced T cells were cocultured with PBMCs of R/R-AML patients according to E:T (1:1, 2:1, 4:1) for 4 h at 37 °C. Then, all cells were harvested and washed once with DPBS (BI, Israel) and labelled with fluorescent antibodies against CD45-V510, CD34-PE, CD7-PE-cy7, CD117-BV421, 7-AAD-Percp, and Annexin-V-APC (Biolegend, USA) for 10 min at 4 °C in the dark. Finally, flow cytometry was used for detection (Miltenyi Biotec, Germany).

### Cytokine secretion assay

The AML cell line KG-1a was coincubated in RPMI 1640 with NTR and naturally selected CD7 CAR-T cells at E: T = 1:1. At the end of overnight coculture, the cells were harvested by centrifugation to retain the supernatant. The LEGENDplex Multianalyte flow assay kit (BioLegend, USA) was used according to the instructions, and the data were analysed using LEGENDplex software (BioLegend, USA) as previously described [38].

### Mouse xenograft model

Based on previously reported studies, we designed an AML xenograft model to study the in vivo effects of naturally selected CD7 CAR-T cells [15, 16]. Six- to 8-week-old nonobese diabetic (NOD)–Prkdcscid-Il2rgem1 (NTG) female mice were purchased from SPF (Beijing) Biotechnology Co. (China) and cared for at the Animal Center of the Fourth Hospital of Hebei Medical University. All in vivo studies were performed in compliance with the Animal Ethics Committee of the Fourth Hospital of Hebei Medical University. The MOLM-13-GFP-luciferase reporter cell line (MOLM-13-GFP-Luc) was constructed to assess in vivo tumour kinetics. Mice were injected with 5 × 10^5^ MOLM-13-GFP-Luc cells through the caudal vein, and the monitoring was performed every 5 days. The IVIS imaging system was used to monitor the construction of acute myeloid leukaemia models. The AML model was successfully constructed. Then, the CD7 CAR group was injected with 1 × 10^6^ CAR^+^ T-cells. The NTR group was injected with the same number of nontransduced T cells, and the vehicle group was injected with the same dose of DPBS (BI, Israel). The tumour burden of mice injected intraperitoneally with 150 mg/kg of D-luciferase at the indicated time was monitored using the IVIS imaging system (Berthold LB983 NC100, Germany) by recording bioluminescence.

### Statistical analysis

The data are presented as the means ± SEMs. Statistically significant differences between samples was determined by an unpaired two-tailed Student’s *t test* and in multiple comparisons by a one-way ANOVA with postTukey’s tests. If the variance was not equal, then the unpaired two-tailed Student’s *t test* with Welch’s correction was employed. All *P* values were calculated using Prism 8 software (GraphPad). The significance of the results are defined as follows: ns, not significant, *p* > 0.05; *p < 0.05; **p < 0.01; and ***p < 0.001 and ****p < 0.0001.

## Results

### CD7 is expressed in R/R-AML patients with minimal residual disease (MRD) but not on normal myeloid cells

**Fig. 1:**
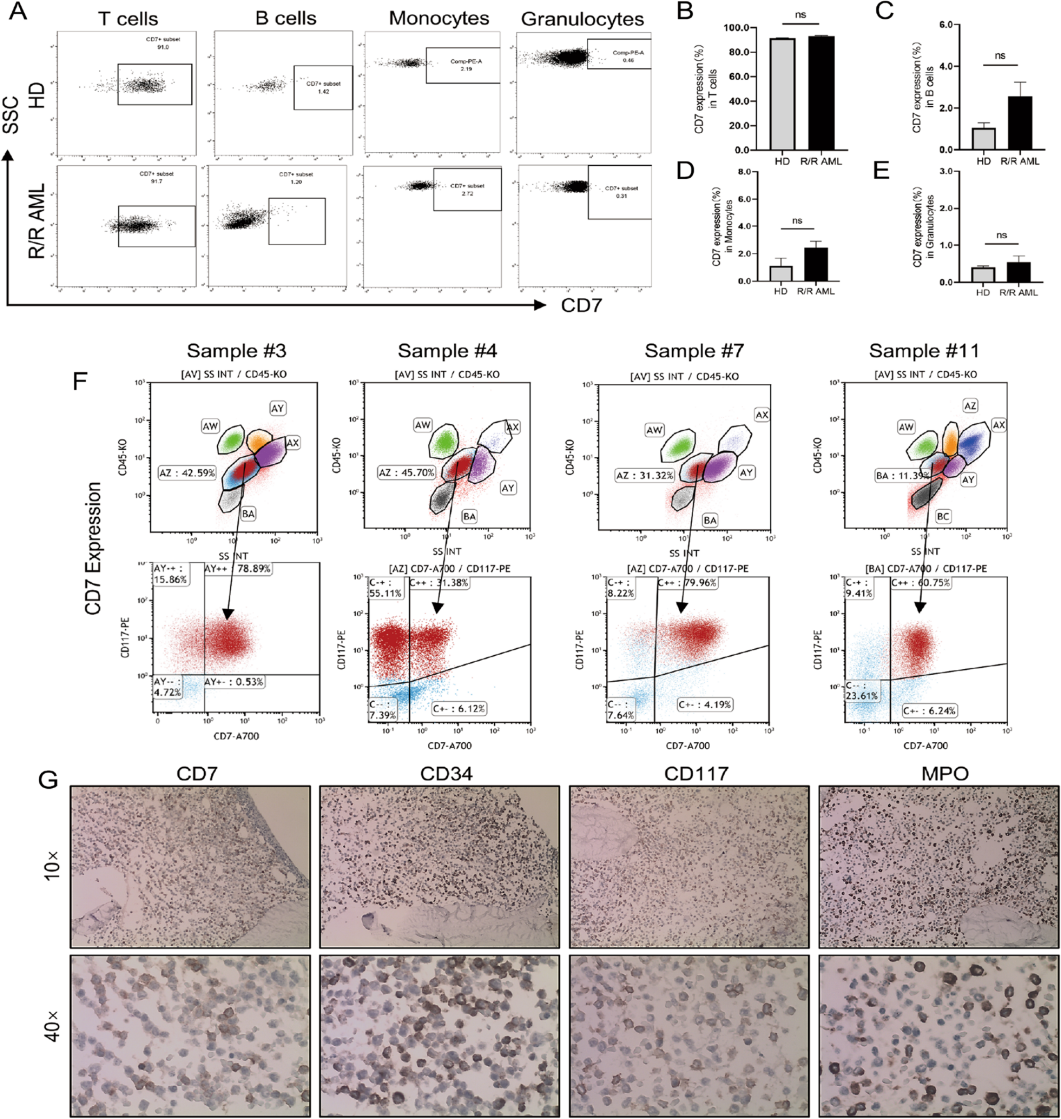
CD7 expression in healthy donors and R/R AML patients **A** CD7 expression in normal immune cells of peripheral blood from healthy donors and AML patients. **B–E** Histogram of CD7 expression analysis in T cells, B cells, monocytes, and neutrophils from R/R AML patients and healthy donors (HDs) (n = 3). **F** FCM was used to determine the percentage of CD7 expression in AML patients with microscopic residual disease (MRD). **G** Images of CD7 expression based on immunohistochemistry of bone marrow biopsies from R/R AML patients. ns, not significant

The distribution of CD7 expression determines the antitumour effect and off-target toxicity of CD7 CAR-T cells. We examined CD7 expression in T cells, B cells, monocytes, and neutrophils from normal cell subsets from HDs. CD7 was highly expressed in T-cell subsets but expressed at low levels or absent in other myeloid subsets. CD7 expression was further determined in normal cell subsets from R/R AML patients and showed similar expression to HDs (Fig. 1A–E). We summarized the expression of CD7 in the MRD-positive reports of R/R-AML patients from July 1, 2022 to January 1, 2021 in the Department of Hematology, the Second Hospital of Hebei Medical University. The results suggested that 5 of the 16 MRD-positive patients (31.25%) exhibited different proportions of CD7 expression, and this finding was also demonstrated by immunohistochemistry (Fig. 1F–G) (Table 1). These results support the use of CD7 as a therapeutic target.

**Table 1:** characteristics of R/R AML

Sample ID	Age	Sex	CD7 +	Cytogenetics
1	42	F	Neg	46, XX
2	57	M	Neg	46, XY
3	73	F	79.78%	46, XX
4	67	M	31.38%	46, XY
5	32	F	Neg	46, XX
6	64	F	Neg	46, XX
7	73	M	79.96%	46, XY, del (9) (q22; q34)
8	30	M	Neg	46, XY, t (8;21) (q22; q22)
9	16	M	Neg	45, X, -Y, t (8;21) (q22; q22)
10	71	F	Neg	46, XX
11	34	F	60.75%	46, XX
12	41	M	Neg	47, XY, + 8
13	59	M	5.00%	46, XY
14	66	M	Neg	46, XY
15	65	F	Neg	Complex
16	37	M	Neg	Complex

### CD7 CAR-T cells exhibit high CAR and negative CD7 expression in vitro

**Fig. 2:**
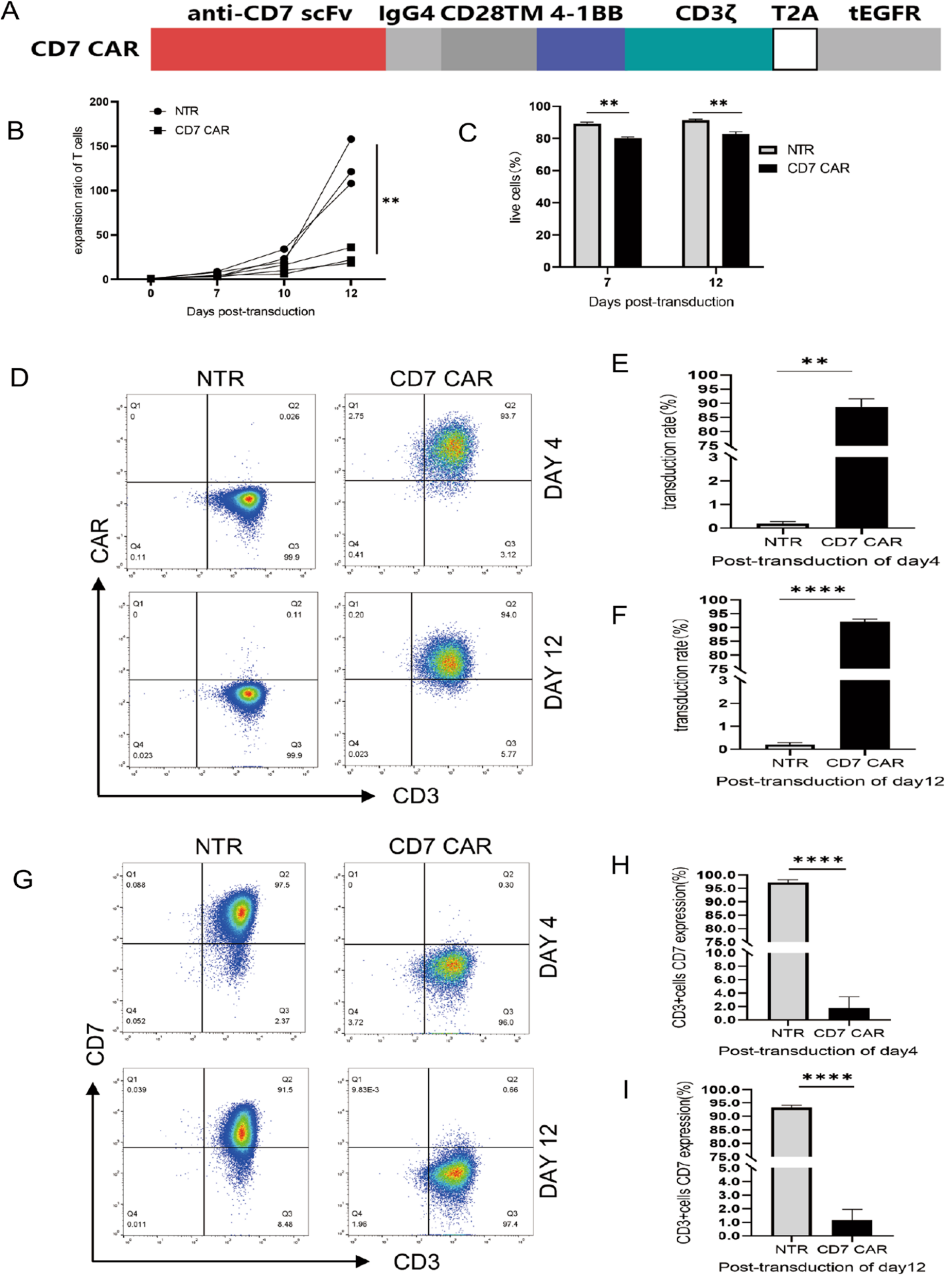
Schematic of the CD7 CAR structure and features of the CD7 CAR in vitro **A** Schematic of the CD7 CAR used in the study. **B** The expansion rate of CD7 CAR-transduced and nontransduced T cells in in vitro culture for 12 days after transduction (n = 3). **C** CD7 CAR-T cell viability at Days 7 and 12 post transduction (n = 3). **D**–**F** Surface expression of CD7 CAR in lentivirus-transduced T cells on Days 4 and 12 post transduction measured by flow cytometry (n = 3). **G**–**I** Surface expression of CD7 in lentivirus-transduced or nontransduced T cells at Days 4 and 12 post transduction measured by flow cytometry (n = 3). NTR, nontransduced. ns, not significant; **P < 0.01; ****P < 0.0001

To determine whether normal T cells can redirect, specifically recognize and attack CD7-expressing AML malignant cells upon loading CD7 CAR. We designed a CAR structure including CD7-specific single-chain variable fragments from CD7 hybridoma antibody TH-69, CD28 transmembrane region, 4–1BB, CD3ζ, and T2A autocleavage sequences as well as endodomain-deleted EGFR (tEGFR) as a CAR expression detection marker for CD7 CAR-T cells [39, 40] (Fig. 2A). We measured the expansion rate of CD7 CAR-T cells at a variety of time points in vitro and found that CD7 CAR-T cells had a lower proliferation ratio than NTR cells (Fig. 2B). Reduced viability of CD7 CAR-T cells was noted compared with nontransduced T cells, but the lowest viability recorded was greater than 78.3% on post transduction Day 7 and 80.7% on post transduction Day 12 (Fig. 2C). We used lentivirus-loaded CD7 CARs to infect normal human T cells and assayed the percentage of CAR^+^ T cells by flow cytometry on post transduction Days 4 and 12. The results indicated that CAR^+^ T cells included a high proportion of CD3^+^ T cells, with the lowest proportion recorded at 83.6% on post transduction Day 4 and 90.8% on Day 12. These values were considerably increased compared with those noted in the control group (Fig. 2D–F). To continuously monitor CD7 expression of CAR-T cells, we measured the percentage of CD7 by flow cytometry on Days 4 and 12 after transduction, and the results showed that CD7 CAR-T cells were mainly dominated by the CD7^−^ T-cell subpopulation either Day 4 or Day 12 after transduction (Fig. 2G–I). As described in previous studies, this finding may imply specific binding of CD7 CAR-T cells to the CD7^+^ cell population, thereby eliminating the CD7^+^ T-cell subpopulation from the CD7 CAR + T-cell population[34].

### Expression of CD7 CAR-T-cell exhaustion markers and cell subpopulation analysis in vitro

**Fig. 3:**
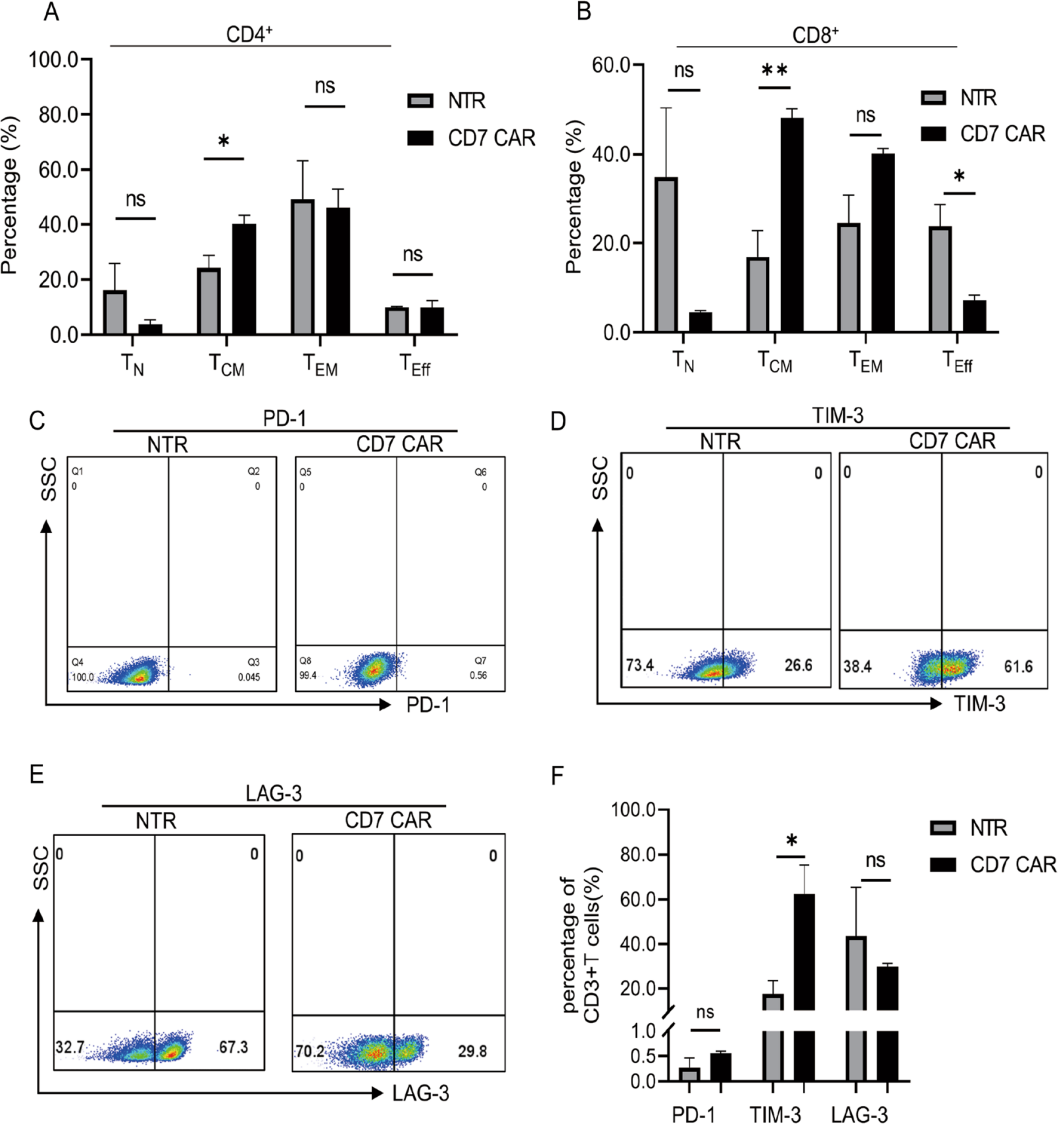
Memory phenotypes of CD7 CAR-T-cell subsets and expression of exhaustion markers in vitro. **A**, **B** Distribution of naïve-like T (T_N_) cells (CD45RO^−^CCR7^+^), central memory-like T (T_CM_) cells (CD45RO^+^CCR7^+^), effector memory-like T (T_EM_) cells (CD45RO^+^CCR7^−^)**,** and effector-like T (T_Eff_) (CD45RO^−^CCR7^−^) of CD7 CAR T or NTR in CD3^+^CD4^+^ or CD3^+^CD8^+^ subsets measured at Day 12 post transduction (n = 3). **C**–**F** Expression of exhaustion markers (PD-1, TIM-3, LAG-3) in CD7 CAR-T cells versus nontransduced T (NTR) cells at Day 12 post transduction (n = 3). ns, not significant; *P < 0.01; **P < 0.01

The memory phenotype of T cells is critical for CAR-T antitumour efficacy and persistence [41, 42]. According to previous studies, T_CM_ cells were defined by CD45RO and CCR7 double positivity [43, 44]. We analysed the cell subpopulations of CD4^+^ T and CD8^+^ T cells of naturally selected CD7 CAR-T and NTR cells, respectively. The results showed that the CD7 CAR and NTR groups did not exhibit differences in the proportion of naïve T cells (T_N_) (CD45RO^−^CCR7^+^) and effector memory T-cells (T_EM_) (CD45RO^+^CCR7^−^) among CD4^+^ and CD8^+^ T-cell subpopulations. However, a significant increase in the proportion of central memory phenotypes (T_CM_) (CD45RO^+^CCR7^+^) was noted in the CD7 CAR group compared to the NTR group. We also failed to observe accelerated terminal differentiation due to the death of naturally selected CD7 CAR-T cells, as there was no difference in effector-like T cells (T_Eff_)(CD45RO^−^CCR7^−^) in the CD4^+^ T-cell subpopulation. Even in the CD8^+^ T-cell subpopulation, the proportion of T_Eff_ was lower in the CD7 CAR group than in the NTR group (Fig. 3A, B).

In addition, we assessed depletion markers in naturally selected CD7 CAR-T cells, and the results suggested a trend of increased programmed cell death protein 1 (PD-1) expression in the CD7 CAR group but, no significant difference was noted compared with the NTR group. Increased T-cell immunoglobulin and mucin domain containing-3 (TIM-3) expression was noted and significantly different from that noted in the NTR group. LAG-3 expression in the CD7 CAR group was not significantly different from that noted in the NTR group, but a decreasing tendency was observed (Fig. 3C–F).

### Evidence of the antitumor function of CD7 CAR-T in vitro

**Fig. 4:**
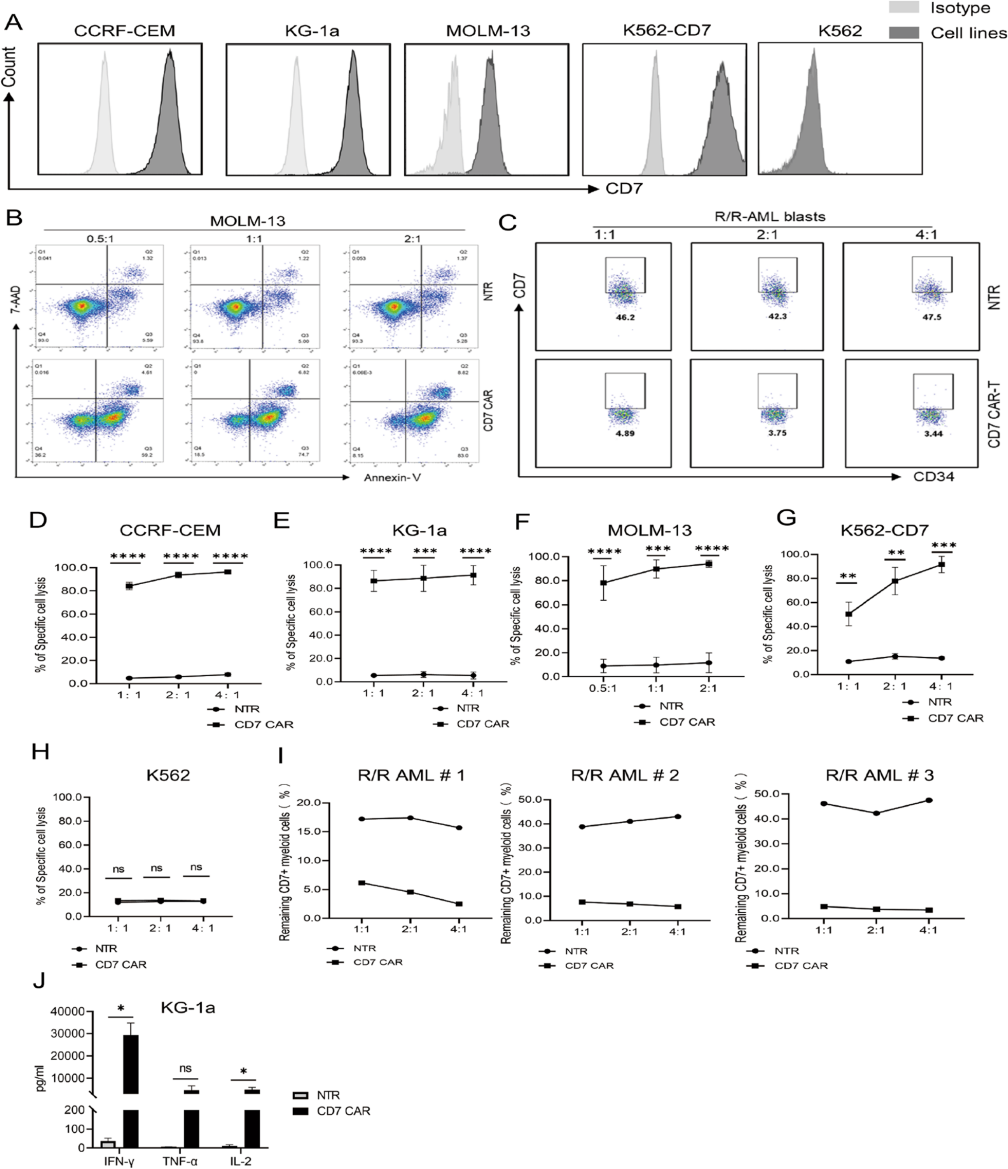
CD7 CAR-T cells exhibit powerful antitumour functions in vitro **A** CD7 expression levels in CCRF-CEM, KG-1a, MOLM-13, K562, and K562-CD7 cells. **B**, **D**–**H** Specific lysis of CCRF-CEM, KG-1a, MOLM-13, K562-CD7, and K562 cells by CD7 CAR-T cells and NTR at various E:T ratios (n = 3). **C**, **I** Cytotoxic effects of CD7 CAR or NTR on primary CD7^+^ R/R-AML blasts at E:T ratios of 1:1, 2:1, and 4:1 (n = 3). **J** Cytokine secretion (IFN-γ, TNF-α, and IL-2) of CD7 CAR or NTR on KG-1a cells at E:T (1:1) (n = 3). ns, not significant; *P < 0.01; **P < 0.01; ***P < 0.001; ****P < 0.0001

The target specificity of CAR-T allows it to kill target-positive cell populations [45]. We chose to measure the CD7 expression levels in the acute myeloid leukaemia cell lines MOLM-13, KG-1a, and K562; CD7-overexpressing K562 (K562-CD7) cells; and the CD7-positive human T-lymphocyte leukaemia cell line CCRF-CEM. The results showed that all cell lines expressed CD7, except K562 (Fig. 4A). To verify the antileukaemic effect of CD7 CAR-T cells in vitro, we used the above cell lines as target cells and set different E:T ratios. We found that CD7 CAR-T cells could rapidly eliminate CD7^+^ cell lines at various dose ratios (Fig. 4B, D–H).

To further validate the cytotoxicity of naturally selected CD7 CAR-T cells against R/R-AML, CD7 CAR-T cells or nontransduced T cells were cocultured with 3 samples from R/R-AML patients. We found that after 4 h of coculture, CD7^+^ AML blasts were significantly reduced in the CD7 CAR-T group compared to the NTR group at any E:T ratio (Fig. 4C, I).

In addition, the naturally selected CD7 CAR-T cells exhibited robust IL-2 and IFN-γ secretion in response to coculture with CD7^+^ AML cell line KG-1a compared to the NTR group (Fig. 4J).

These results demonstrate the potent cytotoxic effect of naturally selected CD7 CAR-T cells on CD7-positive AML cells in vitro.

### CD7 CAR-T cells demonstrated excellent antileukaemic properties in vivo

**Fig. 5:**
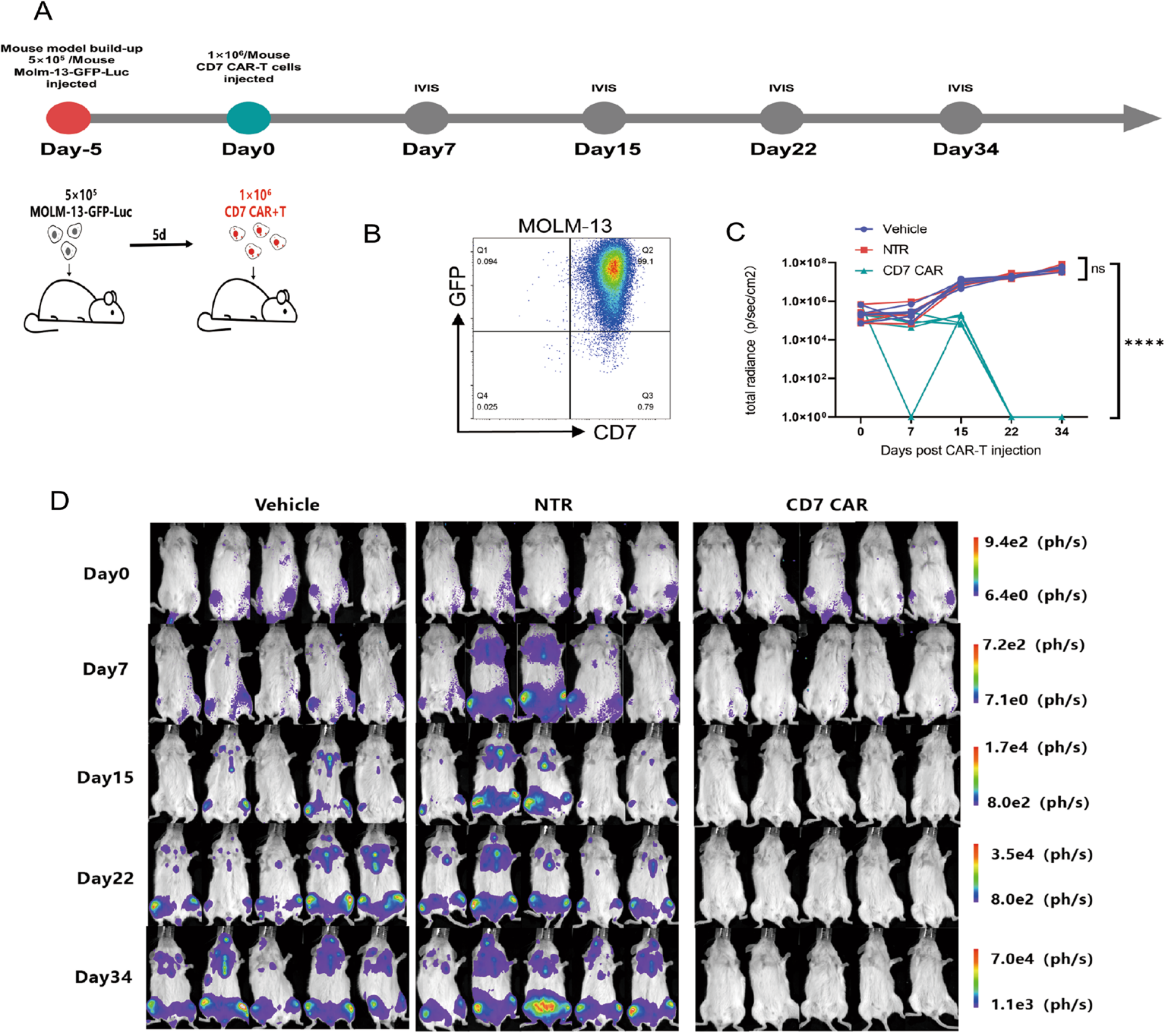
CD7 CAR-T cells exhibit remarkable antileukaemic effects in a leukaemia mouse model **A** Diagram of mouse model construction, CAR-T-cell treatment, and observation timeline. NTG mice received GFP-FFLuc-expressing MOLM-13 cells. After 5 days, different groups of mice received a single intravenous injection of vehicle (DPBS), NTR, or CD7 CAR-T cells, and tumour progression was continuously monitored. **B** GFP-FFLuc-expressing MOLM-13 cells were detected by flow cytometry before infusion. **C** Kinetics of leukaemia progression in individual mice receiving vehicle, NTR or CD7 CAR-T cells by IVIS imaging (n = 5). **D** IVIS images of individual mice showing the progression of leukaemia in the various groups (n = 5). ns, not significant; ****P < 0.0001

Previous in vitro studies have demonstrated the potent antitumour ability of CD7 CAR-T cells. To further validate the clearance of acute myeloid leukaemia cells by CD7 CAR-T cells in vivo. We injected 5 × 10^5^ MOLM-13-GFP-Luc cells into the tail vein of nonobese diabetic (NOD)–Prkdcscid-Il2rgem1 (NTG) mice to generate a xenograft model. IVIS imaging was performed every 5 days after tumour cell infusion to detect tumour implantation, and successful AML implantation was detected at the end of the first 5-day period. Subsequently, 1 × 10^6^ CD7 CAR-T cells or NTR cells were injected, whereas the vehicle group was injected with an equal dose of DPBS buffer (Fig. 5A, B). No mice died during the 34 day monitoring period, and no evidence of graft-versus-host disease (GVHD) was observed. Compared to the NTR group, bioluminescence imaging (BLI) data indicated suppression of leukaemic cells on Day 7 after CD7 CAR-T injection in the CD7 CAR group followed by a rapid decrease in tumour burden, which could not be detected in mice of the CD7 CAR group at 22 days (Fig. 5B, C).

## Discussion

Acute myeloid leukaemia (AML), a heterogeneous disease associated with a wide range of molecular alterations, requires multiple therapeutic strategies that act in synergy to limit disease progression. As an emerging immunotherapeutic tool, chimeric antigen receptor T-cell (CAR-T) therapy is often used as a strategy to reduce residual chemoresistant tumours in patients with acute myeloid leukaemia., i.e., performed before allogeneic haematopoietic stem cell transplantation (allo-HSCT) to minimize the relapse rate post allo-HSCT and avoid severe side effects [45]. Clinical trials of CAR-T targeting CD19 in adults with relapsed or refractory diffuse large B-cell lymphoma have achieved high rates of durable responses, demonstrating the enormous potential of CART-T therapy in the treatment of haematologic malignancies [46]. In the field of acute myeloid leukaemia treatment, CAR-T therapy is also associated with some challenges. Although CAR-T therapies targeting CD123 and CD33 are already in clinical trials, these candidate targets are also frequently found in haematopoietic stem cells, posing risks associated with potential long-term or permanent myelosuppression [14, 21, 47]. In this study, we further explored the availability of naturally selected CD7 CAR-T cells in the treatment of R/R-AML and demonstrated their great antileukaemia ability in vitro and in a xenograft mouse model.

CD7, which has been shown to be expressed in approximately 20–35% of AML patients, is associated with multiple disparate prognoses [18–22, 48]. Similarly, we demonstrated this finding in a subset of 16 R/R AML patients (5/16, 31.25%) with minimal residual disease (MRD). Similar to previous studies, both R/R AML patients and HDs had high expression of CD7 in their normal cell populations, such as NK cells and T cells. In contrast, B cells, monocytes, and neutrophils did not express CD7 [15, 49]. The lack of CD7 expression in myeloid cells prevents the killing of myeloid cells.

CD7-positive T-cells cultured with CD7 CAR-T cells lead to diminished proliferation and increased cell death, as demonstrated in our study and previous studies [15]. Despite the diminished proliferation, our previous study demonstrated that the required dose of naturally selected CD7 CAR-T cells for transfusion back to patients could be achieved [34].

In addition, similar to our previous report, CD7 CAR-T cells were dominated by CD7-negative subpopulations on Day 12 post transduction possibly due to antigenic masking/intracellular sequestration by CD7 CAR [34]. This finding suggests that the naturally selected CD7 CAR-T cells remained CD7 negative before they were administered back to the patients.

Finally, CD7 was positively expressed on T cells and NK cells, which are important components of the human immune system [49, 50]. Previous studies have shown that CD7 CAR-T cells cause defects in CD7^+^ T cells in recipients, but CD7^−^ T-cell subsets appear to replace their function to some extent, thereby alleviating treatment-related T-cell immunodeficiency. In addition, CD7^+^ T-cell populations and NK cell populations were restored after bridging allogeneic haematopoietic stem cell transplantation [30, 34]. Compared with other myeloid-targeted CAR-T cells, CD7 CAR-T cells can avoid damage to normal myeloid cells and reduce haematopoietic toxicity to a certain extent because CD7 is not expressed on other myeloid cells.

Persistence, a key factor affecting CAR-T-cell efficacy, has been widely investigated in previous studies, and it has been shown that the T-cell memory phenotype subpopulation is an important factor in maintaining CAR-T-cell persistence [41, 42]. In the present study, we demonstrated that both CD4^+^ and CD8^+^ T-cell subpopulations exhibited a higher percentage of cells with central memory phenotypes in the naturally selected CD7 CAR-T cells compared with the NTR group, which facilitated a more durable effect of CD7 CAR-T cells. In addition, there was no evidence of accelerated terminal differentiation, even in the CD8^+^ T-cell subpopulation, and the proportion of T_Eff_ with naturally selected CD7 CAR-T cells decreased. The above study demonstrated the persistence of naturally selected CD7 CAR T cells in the in vitro phase of the study. However, further validation of its durability in comparison with other reported CD7 CAR-T cells and in clinical trials of R/R-AML needs to be performed [15, 51–54].

Naturally selected CD7 CAR-T exhaustion marker assays were performed, and the results suggested a trend or statistically significant increase in both PD-1 and TIM-3 expression. In a previous clinical report of T-ALL/LBL, TIM-3 and PD-1 expression levels were not significantly different in patients who achieved CR compared to those who achieved less than CR [34]. Therefore, the impact of increased exhaustion markers on naturally selected CD7 CAR-T cells for R/R-AML needs to be validated by further clinical trials and long-term observations.

In addition, CD7^+^ cell lines and primary AML blasts from R/R-AML patients in vitro and CD7^+^AML xenograft models were used to assess the antileukaemic ability of naturally selected CD7 CAR-T cells, demonstrating powerful cytotoxic effects on CD7^+^ AML cells.

Although our proposed naturally selected CD7 CAR-Ts exhibit diminished proliferation due to cell death at the in vitro culture stage, the CAR-T dose for patient transfusion can still be achieved. In addition, CD7 CAR-Ts may be less costly and exhibit lower risks associated with gene editing. In addition, in recent years, the construction of CAR-T cells by isolating CD7-negative cell populations or the use of ibrutinib and dasatinib to inhibit fratricide has demonstrated good potential value [52, 54]. Hai-Ping Dai et al. demonstrated the safety and efficacy of CD7 CAR-T cells using protein expression blockers to block CD7 expression at the CAR-T-cell membrane for the treatment of R/R early T-cell precursor lymphoblastic leukaemia/lymphoma (ETP-ALL/LBL) in patients with TP53 mutations [51]. Yongxian Hu et al. investigated allogeneic CD7 CAR-T cells for R/R CD7-positive haematologic malignancies, including one patient with CD7-positive acute myeloid leukaemia and 11 patients with T-cell leukaemia/lymphoma. This study demonstrated the safety and efficacy of allogeneic CD7 CAR-T cells for CD7-positive haematologic malignancies [53]. Both the abovementioned studies and our proposed naturally selected CD7 CAR-T cells have explored the use of CD7 CAR-T cells in the treatment of haematologic malignancies. However, it seems that CAR-T cells targeting CD7 show different characteristics in clinical trials compared with CAR-T cells targeting CD19. Thus, the actual performance of CD7 CAR-T cells in the treatment of R/R-AML patients should be assessed in further clinical trials and long-term clinical observations [55].

In conclusion, naturally selected CD7 CAR-T cells, as a CAR-T treatment strategy without additional treatments, such as CD7 knockdown, can reduce the cost and additional unknown risks associated with gene knockdown to some extent. Patients can benefit from avoiding the risk of additional gene knockouts and reduced production costs of naturally selected CD7 CAR-T cells as a bridging allogeneic HSCT pretreatment for R/R-AML. This study demonstrated the feasibility of naturally selected CD7 CAR-T cells in the treatment of patients with CD7^+^ R/R-AML in the preclinical study phase. However, R/R-AML is a complex disease type, and the effectiveness of naturally selected CD7 CAR-T therapy needs to be further tested in clinical trials.

## Data Availability

The original contributions presented in the study are included in the article/Supplementary Material, further inquiries can be directed to the corresponding authors.

## Abbreviations

R/R AML: Relapsed and refractory acute myeloid leukemia
CAR-T: Chimeric antigen receptor T cell
MRD: Minimal residual disease
HD: Healthy donor
HSCT: Hematopoietic stem cell transplantation
NTR: Non-transduced
CR: Complete remission
PBMCs: Peripheral blood mononuclear cells
